# Multimodal Approach of Pulmonary Artery Intimal Sarcoma: A Single-Institution Experience

**DOI:** 10.1155/2017/7941432

**Published:** 2017-08-20

**Authors:** S. Secondino, V. Grazioli, F. Valentino, M. Pin, A. Pagani, A. Sciortino, C. Klersy, M. G. Callegari, P. Morbini, R. Dore, M. Paulli, P. Pedrazzoli, A. M. D'armini

**Affiliations:** ^1^Division of Medical Oncology, Fondazione IRCCS Policlinico San Matteo, Pavia, Italy; ^2^Cardiothoracic Surgery Department, Fondazione IRCCS Policlinico San Matteo and Pavia University School of Medicine, Pavia, Italy; ^3^Service of Biometry & Statistics, Fondazione IRCCS Policlinico San Matteo, Pavia, Italy; ^4^Unit of Pulmonary Rehabilitation, ICS Maugeri Istituto Scientifico di Montescano, Pavia, Italy; ^5^Unit of Pathology, Fondazione IRCCS Policlinico San Matteo and Pavia University School of Medicine, Pavia, Italy; ^6^Division of Radiology, Fondazione IRCCS Policlinico San Matteo, Pavia, Italy

## Abstract

**Introduction:**

Pulmonary artery sarcoma (PAS) is a rare tumor, whose therapeutic approach is mainly based on surgery, either pneumonectomy or pulmonary endarterectomy (PEA). The prognosis reported in published series is very poor, with survival of 1.5 months without any kind of treatment.

**Patients and Methods:**

From January 2010 to January 2016, 1027 patients were referred to our hospital for symptoms of acute or chronic pulmonary thromboembolic disease. Twelve patients having a confirmed diagnosis of PAS underwent PEA. Median age was 64.5 years. Most patients had a long history of symptoms, having a median time of 7.5 months from onset of symptoms to surgery.

**Results:**

Following PEA and cardiopulmonary rehabilitation, 10 patients received conventional chemotherapy with doxorubicin and ifosfamide, starting at a median of 42 days from surgery. Four patients also received radiotherapy. Four patients have died due to disease progression, while 7 are still alive, with 5 being disease-free at 4–55+ months from diagnosis.

**Conclusions:**

In patients with PAS, a multimodal approach including PEA, CT, and RT is feasible but it should be evaluated individually, according to the tumor extension and the patient's clinical condition. Apart from improving quality of life mainly by reducing or delaying symptoms due to PH, it may improve life expectancy.

## 1. Introduction

Pulmonary artery sarcoma (PAS) was first described in 1923 [1]. Since that time, about 300 PAS have been reported in the English-language literature, as case reports or small series. Primary PAS is the most frequent sarcoma of the great arteries [2], in most cases arising in the area derived from the embryologic bulbus cordis, which leads to PAS predominantly occurring in the main pulmonary artery [3] which is involved in 80% of cases: the left pulmonary artery in 58%, the right pulmonary artery in 57%, both arteries in 37%, the pulmonary valve in 29%, and the right ventricle in 8%.

Primary PAS are classified by location as either luminal or mural in origin and then further subclassified histologically. Luminal PAS are thought to derive from pluripotential mesenchymal cells of the intima, while mural sarcomas, less frequent than luminal sarcomas, can be difficult to distinguish from sarcomas of the lung parenchyma due to their growing; they are more likely to appear as mass-like lesions [4]. PAS typically affect middle-aged people, favoring women slightly. Common symptoms at time of presentation often mirror those of pulmonary embolism, including progressive dyspnea, cough, and chest pain [3]. However, symptoms onset is usually more gradual with PAS than pulmonary embolism.

Given the rarity of PAS, only case reports and small series have been published, with the majority focusing on histopathological features and surgical management of the disease [4–7]. Surgery remains the mainstay of management of patients with PAS and can include pulmonary endarterectomy (PEA), lobectomy, or pneumonectomy, based on the extension of the disease and patient clinical conditions [5, 7, 8]. The prognosis reported in published study is extremely poor, with survival of 1.5 months without surgical resection and 17 months in case of surgery [7–10]. The role of additional chemotherapy (CT) and radiotherapy (RT) after surgical resections remains largely unproven.

In this study, we report our single-institution experience concerning the management of 12 consecutive patients with PAS who were referred to our institution over a period of 6 years period and have been treated with PEA and subsequent CT and RT. A recently published survey has included 20 patients diagnosed over a 14-year period [8], with 14 being treated with PEA.

## 2. Patients and Methods

**Figure 1:**
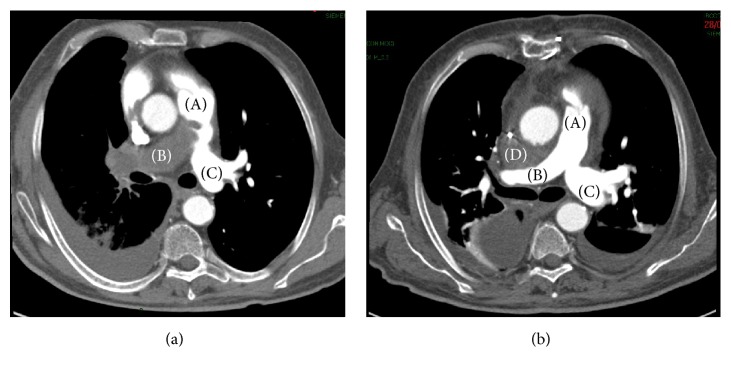
*CT scan of patient number 10*. (a) Presurgical CT scan: right pulmonary artery completely occluded by soft tissue mass (B); proximal aspect of the mass in the pulmonary trunk (A); left pulmonary artery appears preserved (C). (b) Postsurgical CT scan (same level): the intra-arterial mass has been completely removed. Small mediastinal haematoma (D) between ascending aorta and right pulmonary artery.

Among 1027 patients referred to our Cardiac Surgery Unit for suspected chronic pulmonary hypertension (PH) potentially requiring PEA from January 2010 to January 2016, 574 had a confirmed PH and 448 underwent TEAP. Twenty-two patients had a radiological diagnosis of suspected tumor growing in the pulmonary artery by high-resolution CT scan of the thorax, which is performed within a conventional work-up for chronic thromboembolic PI. CT scan revealed soft tissue masses (Figure 1) occluding the artery; it demonstrated high- or low-attenuation areas consistent with hemorrhage or necrosis, soft tissue density, and variable contrast enhancement, thus suggesting a tumor mass over thrombus [3]. Two patients (numbers 3 and 11) underwent also FDG-PET, resulting in a significant FDG uptake.

All these patients had symptoms of acute or chronic thromboembolic disease. Among 22 patients with radiological diagnosis of tumor, 20 were eligible for surgery: eight patients had metastatic involvement of the pulmonary artery, arising in other sites not yet diagnosed. Twelve patients had PAS at pathology evaluation.

Two patients were referred for chemotherapy due to the presence of nonresectable tumor mass.

The median age of patients undergoing PEA for PAS was 64.5 years (range: 32–84); 9 patients were female. 9 patients had a bilateral disease and 3 had a metastatic disease involving the lung. In most cases (8 patients), the disease involved the main pulmonary artery. Nine patients had pulmonary hypertension (PH), with two with severe hemodynamic instability requiring emergency surgical treatment. Most patients had a long history of symptoms due to pulmonary hypertension, with the median time between onset of symptoms and surgery being 7.5 months (range: 2–33). Relevant patient characteristics are summarized in Table 1. All patients had given their written informed consent to use their data, as approved by the Institutional Ethical Board.

### 2.1. Surgery

All patients underwent PEA, using a technique identical to that adopted for chronic thromboembolic pulmonary hypertension. As previously described [11], the surgical approach is a median sternotomy and PEA is performed using cardiopulmonary bypass (CPB) and moderate hypothermic ventricular fibrillation. The main pulmonary arteries are sectioned longitudinally, and the dissection plane is accurately identified. Our surgical technique has been the same as that reported in published studies, with some technical changes (e.g., the aorta is left unclamped to reduce the potential risk of embolization of aortic atherosclerotic plaques and myocardial protection relies on hypothermia, left ventricular venting, and frequent reperfusion sections) [12]. The cerebral protection strategy has changed over time mainly to reduce the burden of hypothermic circulatory arrest and currently consists of short periods (7–10 minutes) of moderate hypothermic circulatory arrest followed by short periods (≥5 minutes) of reperfusion, with monitoring using cerebral near-infrared spectroscopy to determine the length of each reperfusion period. At the end of PEA, the left ventricular vent is clamped, and bronchoscopy is performed to verify the absence of airway bleeding. After weaning from CPB, the pericardium is closed to reduce the formation of adhesions and improve postoperative right ventricular function [12].

**Figure 2:**
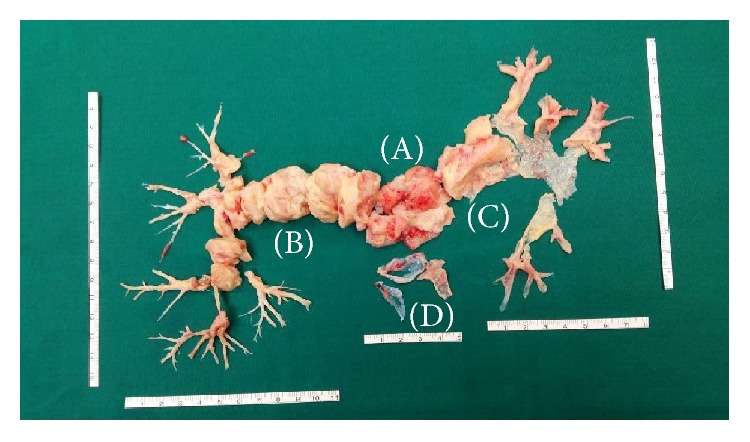
Pathological specimen after pulmonary endarterectomy of patient number 10. There is evidence of the tumor growth into the pulmonary artery, particularly the pulmonary trunk (A), the right pulmonary artery (B), and the left pulmonary artery (C); peeling of the pulmonary valve (D).

Pathology evaluation revealed intimal sarcoma of pulmonary artery, being undifferentiated in 7 cases (Table 2). Three patients had pulmonary valve involvement requiring pulmonary valve replacement in two cases and peeling with curettage in one case (Figure 2). One patient (number 12) died after surgery due to cardiovascular complications. After PEA, all patients had a short course of cardiopulmonary rehabilitation.

One patient (number 6) underwent surgical resection of single-lung metastasis 30 days after PEA.

### 2.2. Chemotherapy and Radiotherapy

Following surgery and cardiologic workup, eligible patients received conventional CT with doxorubicin (20 mg/mq a day, for three consecutive days) and ifosfamide (3000 mg/mq a day, for three consecutive days) for up to six cycles as adjuvant treatment of both local disease and metastatic cancer.

From 2014 onwards, the protocol was modified based on published evidence [7] and patients with local disease were given 4 cycles of the same CT regimen followed by intensity modulated RT (IMRT) at a radical dose of 60 Gy in 30 fractions to surgical bed.

Follow-up after completion of the multimodal approach consisted of high-resolution CT scan of the thorax and CT scan of the abdomen every 4 months during the first year and every 6 months thereafter.

### 2.3. Statistics

Data were described with median and range if they were continuous and counts and % if they were categorical. The Kaplan-Meier cumulative survival was computed and plotted; median survival was reported. Median follow-up (25th–75th percentiles) was computed with the inverse Kaplan-Meier method. Stata 14 (College Station, TX, USA) was used for computation.

## 3. Results

The median length of hospital stay for surgery was 13 days (range: 10–22). One patient (number 1) developed neurological defects due to cerebral anoxia requiring long-lasting neurological rehabilitation with partial recovery (lasting memory defects). Atrial fibrillation and anemia requiring blood transfusion were observed in two patients (numbers 5 and 10): one also having haemothorax (patient number 10) and one with mild renal failure (number 5).

Notwithstanding the cardiac surgery, no patient had cardiac dysfunction contraindicating the administration of anthracycline-containing CT.

Ten patients received CT with doxorubicin and ifosfamide starting at a median of 42 days (range: 22–69) after surgery. Nine have completed the planned schedule of CT, receiving a median of 5 cycles (range: 4–6); one patient received only 2 cycles due to early evidence of disease progression. One patient (number 1) did not receive postsurgery CT due to neurologic complications occurring in the rehabilitation phase and was treated at disease recurrence 10 months after PEA.

Overall CT was well tolerated; grade III/IV neutropenia (prophylactic G-CSF was administered in all patients) was observed in 2 patients and grade II mucositis in 1 patient. One patient (number 7) required hospital admission for reversible encephalopathy due to ifosfamide. Four patients underwent IMRT at the end of the CT program, starting at a median of 49 days (range: 49–63) from the end of chemotherapy. With a median follow-up of 21 months, no cardiac complications have been observed.

**Figure 3:**
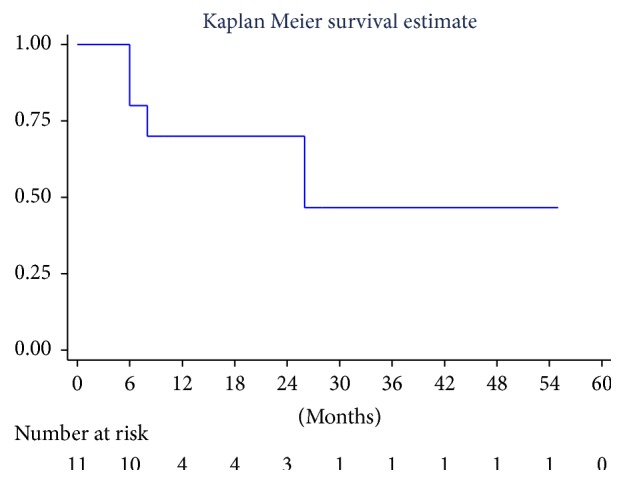
*Overall survival of patients with pulmonary artery sarcoma*. Kaplan-Meier survival estimate for patients in the series (*n* = 11), except one who died after surgery.

The median follow-up after surgery was 21 months (25th–75th percentiles, 10–28 months). Four patients have died due to disease progression at 6, 6, 8, and 24 months from surgery, while 7 patients are still alive, with 5 being disease-free at 4, 10, 21, 28, and 55 months from diagnosis. The median survival time was 26 months (Figure 3).

## 4. Discussion

PAS is a rare disease mimicking acute or chronic thromboembolic disease often causing delay in diagnosis and thus surgical and medical treatment [5, 7, 13–15]. Some specific characteristics can lead to correct differential diagnosis, including the absence of clinical status improvement or mass size reduction after anticoagulant therapy, the absence of thrombophilic risk factors, and the bulky central angiographic presentation [5, 9, 14–16]. However, in our experience, including the present series, patients have been diagnosed with PAS in most cases after being referred for long-lasting PH thought to be of thrombotic origin and therefore potentially requiring PEA. All radiological diagnoses of PAS by high-resolution CT scan of the thorax were confirmed at surgery and pathological examination. We previously reported [5] that PH is a sign of bilateral disease, even in case of a unilateral CT pattern. This is confirmed by the present series as ten out of twelve patients had PH (median: 65 mmHg; range: 45–100 mmHg) and all of them had confirmed bilateral disease at surgery.

Two patients investigated by FDG-PET had significant FDG uptake. Several authors have reported that FDG-PET has been useful in diagnostic workup for differentiating among or staging malignant diseases and monitoring the response to treatment [17, 18]. Among unusual findings, other authors reported case reports of patients with PAS with a lack of uptake of FDG in PET [19, 20]. Therefore, the role of this procedure in the diagnostic workup of PAS remains controversial and requires further investigation.

The prognosis of patients with PAS is generally poor. Surgery, either PEA or lobectomy/pneumonectomy, increases survival and ameliorates symptoms associated with pulmonary artery occlusion, thus allowing patients to receive additional forms of systemic or local therapies [7, 8, 21]. PEA, when feasible, should be considered the treatment of choice. In our series, it provided complete macroscopic tumor removal in most cases and was also effective with palliative intent; eleven out of twelve patients, after rapid recovery of symptoms and heart function improvement, were eligible to receive conventional anthracycline-containing CT with limited side effects. Radiotherapy was also administered after completion of CT in selected patients.

As PAS remains a rare disease, limited data are available on the role of postsurgery treatment. Among 31 patients receiving any surgical procedure [7], 4 died in the postsurgery phase and 15, 2, and 1 underwent CT, RT, or both, respectively. In a more recently published series of 14 patients undergoing PEA [8], there were three perioperative deaths, five patients received postsurgical CT, and, after completion, four also had RT: one of them in adjuvant setting and three for metastatic disease. The CT regimen was different: 3 patients received anthracycline, 1 ifosfamide alone, and 1 the combination of both. Overall, the two studies indicate a trend towards better survival for patients who received postoperative chemotherapy and/or RT compared to those who had surgery alone.

Because of the shorter follow-up and the limited number of patients in our series and although this is one of the largest single-center experiences of PAS treated with a defined multidisciplinary approach, we cannot definitively prove that the addition of CT and RT considerably extend life expectancy over surgery alone. However, survival curves appear to match favorably with previous reports of PAS patients undergoing surgery alone [7, 8, 10, 14].

In summary, while early diagnosis is an essential prerequisite to allow for optimal management of PAS, it is of pivotal importance that patients with suspected diagnosis of PAS be promptly referred to expert centers for PEA where a multidisciplinary team is available. As shown in our study, PEA represents a more effective and the safest surgical procedure. It should be preferred also because it represents a rapid multidisciplinary pathway of care, along with CT and RT. Further clinical research is mandatory to improve the outcome of patients and for a better understanding of the role of systemic anticancer therapy and RT.

## Figures and Tables

**Table 1:** Patient characteristics (*N* = 12).

Variables	Number of patients (%)
Gender	
(i) Female	9 (75%)
(ii) Male	3 (25%)
Median age	64,5 (range: 32–84)
(i) Male	77 (range: 32–84)
(ii) Female	64 (range: 37–77)
Symptoms	
(i) Dyspnea	10 (83.3%)
(ii) Chest pain	7 (58.3%)
(iii) Cough	2 (16.6%)
(iv) Hemoptysis	2 (16.6%)
(v) Fever	2 (16.6%)
(vi) Cardiovascular event	2 (16.6%)
Dyspnea WHO classification	
(i) II	7 (58.3%)
(ii) III	2 (16.6%)
(iii) IV	3 (25%)
Pulmonary hypertension	
(i) Mild (25–40 mmH)	0
(ii) Moderate (41–55 mmH)	4 (33.3%)
(iii) Severe (>56 mmH)	6 (50%)
Disease stage	
(i) Local disease	9 (75%)
(ii) Metastatic disease	3 (25%)
Histology grading	
(i) G1	0
(ii) G2	5 (41.6%)
(iii) G3	7 (58.3%)
Hospital stay (days)^*∗*^	
(i) ≤7	0
(ii) >7<15	8 (72.7%)
(iii) ≥15	3 (27.2%)

^*∗*^Excluding the patient who died after PEA due to cardiovascular event.

**Table 2:** Main characteristics and outcomes of the patients.

Patients (*N*)	Sex Age	Symptoms duration (months)	Symptoms	Date of diagnoses	Histologic type (differentiation)/grading	Site(s) of disease	IP (PAPS) mmHg	Treatment after surgery	Time from surgery to CT (days)	*N* cycles of CT	Disease status	Overall survival (months)
1	F 45	9	Dyspnea Right cardiac failure	2010	IS (fibrosarcoma) G2	Local M+ (lung, kidney, nodes)	85	CT^*∗*^	10 months^*∗*^	NA	PD	26^†^
2	F 65	2	Pain Cough	2011	IS (osteocondroid diff) G2	Local (bilateral)	45	CT	50	6	NED	55
3	M 32	9	Fever Pain Dyspnea	2012	IS (undiff) G3	Local (unilateral)	65	CT	22	6	PD	6^†^
4	F 74	8	Dyspnea Pain	2012	IS (fibrous histiocytoma diff) G3	Local (unilateral)	—	CT	48	6	PD	8^†^
5	F 77	7	Dyspnea Pain Cough Hemoptysis	2012	IS (mixofibrous diff) G2	Local M+ (lung)	>60	CT	69	2	PD	6^†^
6	F 49	33	Dyspnea	2013	IS (leiomuscolar diff) G2	Local M+ (lung)	90	CT	42	5	NED	28
7	F 64	15	Dyspnea Pain Dysesthesia	2014	IS (undiff) G3	Local (bilateral)	45	CT/RT	30	4	NED	21
8	M 77	2	Dyspnea Pain	2015	IS (undiff) G3	Local (bilateral)	100	CT/RT	25	4	PR	11
9	F 49	2	Heart failure	2015	IS (undiff) G3	Local (bilateral)	45	CT/RT	36	4	NED	10
10	M 84	4	Dyspnea Pain	2015	IS (undiff) G2	Local (bilateral)	70	CT	44	4	PD	8
11	F 37	6	Dyspnea Hemoptysis	2016	IS (osteosarcoma diff) G3	Local (bilateral)	—	CT	30	4	NED	4

IS: intimal sarcoma; M+: metastatic disease; CT: chemotherapy (adriamycin and ifosfamide); RT: radiotherapy; NA: not applicable; PD: progressive disease; NED: not evidence of disease; PR: partial response. ^†^Patient died. ^*∗*^CT was given at disease progression. The patient who died perioperatively was excluded.
